# Characterizing Human Peripheral Blood Lymphocyte Phenotypes and Their Correlations with Body Composition in Normal-Weight, Overweight, and Obese Healthy Young Adults

**DOI:** 10.3390/medicina60091523

**Published:** 2024-09-18

**Authors:** Irina-Bianca Kosovski, Cristina Nicoleta Ciurea, Dana Ghiga, Naomi-Adina Ciurea, Adina Huțanu, Florina Ioana Gliga, Anca Bacârea

**Affiliations:** 1 Department of Pathophysiology, George Emil Palade University of Medicine, Pharmacy, Science and Technology of Târgu Mureș, 540139 Târgu Mureș, Romania; 2 Doctoral School, George Emil Palade University of Medicine, Pharmacy, Science and Technology of Târgu Mureș, 540139 Târgu Mureș, Romania; 3 Department of Microbiology, George Emil Palade University of Medicine, Pharmacy, Science and Technology of Târgu Mureș, 540139 Târgu Mureș, Romania; 4 Department of Research Methodology, George Emil Palade University of Medicine, Pharmacy, Science and Technology of Târgu Mureș, 540139 Târgu Mureș, Romania; 5 Department of Internal Medicine, George Emil Palade University of Medicine, Pharmacy, Science and Technology of Târgu Mureș, 540139 Târgu Mureș, Romania; 6 Department of Laboratory Medicine, George Emil Palade University of Medicine, Pharmacy, Science and Technology of Târgu Mureș, 540139 Târgu Mureș, Romania; 7 Center for Advanced Medical and Pharmaceutical Research, George Emil Palade University of Medicine, Pharmacy, Science and Technology of Târgu Mureș, 540139 Târgu Mureș, Romania

**Keywords:** obesity, young healthy adults, NK dim, NK bright, peripheral blood lymphocytes, flow cytometry, CD69, inflammation

## Abstract

*Background and Objectives:* Obesity-associated chronic low-grade inflammation supports various systemic alterations. In this descriptive study, 122 apparently healthy adults aged 20 to 35 years were voluntarily included and classified based on body mass index (BMI) as normal-weight (NW), overweight (OW), and obese (OB). This study aims to characterize peripheral blood (PB) lymphocyte (Ly) phenotypes and investigate their correlations with body composition indices (BCIs) in healthy young adults. *Materials and Methods:* The following BCIs were measured: waist circumference, hip circumference, height, waist-to-hip ratio, waist-to-height ratio, total body fat mass, visceral fat level, weight, and BMI. White blood cell count (WBC), Ly absolute count, serum TNF-α, and IFN-γ were quantified. Ly subpopulations were analyzed as follows: total TLy (TTLy—CD45^+^CD3^+^), early activated TLy (EATLy—CD45^+^3^+^69^+^), total NKLy (TNKLy—CD45^+^CD3^−^CD56^+^CD16^+^), NK^dim^ (low expression of CD56^+^), NK^bright^ (high expression of CD56^+^), BLy (CD45^+^CD3^−^CD19^+^), T helper Ly (ThLy—CD45^+^CD3^+^CD4^+^), and T cytotoxic Ly (TcLy—CD45^+^CD3^+^CD8^+^). *Results:* Higher BMI has significantly higher WBC and BLy (*p* < 0.0001; *p* = 0.0085). EATLy significantly decreased from NW to OB (3.10—NW, 1.10—OW, 0.85—OB, *p* < 0.0001). Only EATLy exhibited significant negative correlations with all the BCIs. A significantly higher TNF-α was observed in the OW and OB groups compared to the NW group. IFN-γ increased linearly but nonsignificantly with BMI. TTLy showed a nonsignificant positive correlation with both IFN-γ and TNF-α, while EATLy showed a negative correlation, significant only for IFN-γ. NKLy subpopulations exhibited a consistent negative correlation with TNF-α, significant only for NK^dim^ (*p* = 0.0423), and a nonsignificant consistent positive correlation with IFN-γ. A nonsignificant negative correlation between age and both TNKLy (r = −0.0927) and NK^dim^ (r = −0.0893) cells was found, while a positive correlation was found with NK^bright^ (r = 0.0583). *Conclusions:* In conclusion, the baseline immunological profile of PB is influenced by excessive adipose tissue in healthy young adults.

## 1. Introduction

Obesity is a global health issue associated with a chronic low-grade inflammatory (CLGI) state that predisposes individuals to various secondary pathophysiological changes [1]. The World Health Organization has reported that obesity prevalence is increasing over time. However, the COVID-19 pandemic exacerbated the pre-existing “silent pandemic” of obesity, impacting individuals across all age groups [2,3,4].

Aging is often associated with an increased risk of developing multiple comorbidities. Recently, the concept of immunosenescence has been defined as a continuous process that remodels innate and adaptive immunity, affecting both their quantitative and functional expression [5]. One consequence of this remodeling is “inflammaging”, characterized by an imbalance between pro- and anti-inflammatory processes [5]. When aging is combined with obesity, it creates a significant inflammatory environment that interferes with the baseline status, such as peripheral blood lymphocyte expression [6].

Obesity CLGI is traditionally defined based on the concentration of highly sensitive C-reactive protein (hs-CRP). In healthy adults, it has been observed that hs-CRP levels significantly increase in correlation with various adiposity indices, such as arm circumference, hip circumference, waist circumference, waist-to-hip ratio, waist-to-height ratio, body mass index (BMI), total body fat mass (kg), and visceral fat level [7]. This inflammatory microenvironment predisposes individuals to altered functions and phenotypes of the various immune cells, which are key components of the innate and adaptive immune systems. Changes have been observed in both levels: peripheral blood and tissues [8,9,10,11]. Due to the accessibility of performing various laboratory analyses on hospitalized patients or those attending routine check-ups for different conditions, many studies have focused on investigating immune blood profiles in patients with multiple pathologies. Consequently, the baseline immunological profile in healthy individuals with excessive adipose tissue remains under investigation. Therefore, it is necessary to exclude common sources of inflammatory interference, such as age and possible comorbidities, and specifically focus on the effects caused solely by excessive adipose tissue on the expression of immune cells in peripheral blood.

We propose to study a population of healthy young adults within an age range that is considered the “golden age”, as this period reflects a hormonal balance regarding the maturation process and is early enough to prevent significant effects of immunosenescence. Furthermore, complete blood counts are already a routine test, and flow cytometry is an accessible technique that can relatively quickly and easily provide information regarding lymphocyte populations. Moreover, because an adipose tissue biopsy is an invasive procedure, correlating these findings with anthropometric and body composition indices (adiposity indices) can guide the understanding of how changes in adipose tissue (quantity and distribution) influence lymphocyte expression in peripheral blood, representing the base of future obesity-associated alterations. Thus, this study primarily aims to examine the phenotypic characteristics of peripheral blood lymphocytes and their correlations with body composition in apparently healthy young adults aged 20–35, both with and without excessive adipose tissue. The secondary aims are to investigate (1) the correlation between T lymphocytes and NK subpopulations with IFN-γ and TNF-α and (2) the correlation between age and NK subpopulations.

## 2. Materials and Methods

### 2.1. Study Design and General Information

The Ethics Committee of the County Clinical Emergency Hospital of Târgu Mureș approved this cross-sectional study (Decision no. Ad.29270/8 December 2020), which adheres to the guidelines outlined in the Declaration of Helsinki. Informed written consent was obtained from all subjects. Participation in the study was voluntary and the participants were not hospital patients.

This study included clinically healthy subjects aged between 20 and 35 years, encompassing all BMI ranges (normal weight, overweight, and obese), except for underweight. Participants were voluntarily recruited after the study information was disseminated across various public social platforms and groups, targeting individuals who met the inclusion criteria. Clinical health was defined as the absence of chronic inflammatory diseases, a minimum of 30 days since the last occurrence of any acute inflammatory episode, and no ongoing drug therapy, particularly anti-inflammatory drugs. Pregnant and lactating women were excluded. All data were collected through a questionnaire completed by the subjects after providing informed written consent.

### 2.2. Anthropometric Measurements

The anthropometric measurements were divided into mechanical and electronic measurements. The mechanical measurements were performed using a centimeter and a stadiometer, respectively, for the following parameters: waist circumference (WC, cm), hip circumference (HC, cm), and height (Ht, cm). The waist-to-hip ratio (WHR) and waist-to-height ratio (WHtR) were calculated. For the electronic measurements, the TANITA BC-1000 Inner Scan^®^ scale (TANITA Corporation, Tokyo, Japan) with the bioelectrical impedance technique was utilized to assess total body fat mass (TBFM, kg), visceral fat level (VFL), weight (kg), and BMI.

### 2.3. Laboratory Methods

#### 2.3.1. Blood Sample Collection

The blood samples were collected in the morning following an 8–10 h fasting period. BD Vacutainers^®^ (Becton, Dickinson and Company, Franklin Lakes, NJ, USA) containing K3 EDTA anticoagulant, as well as vacutainers with a clot activator and gel serum separator, were used for hematological (flow cytometry) and immunological tests, respectively. The blood collection took place in the Medical Analysis Laboratory of the County Emergency Clinical Hospital of Târgu Mureș and was performed by trained nurses. After 30 min at room temperature, serum samples were centrifuged, aliquoted, and stored at −80 °Celsius, and hematological tests were immediately performed. For a proper interpretation of the results, all the immunological tests were analyzed on the same day, using the same calibration curve.

#### 2.3.2. Complete Blood Count

White blood cell count (WBC, ×10^3^/µL) and Ly absolute count (×10^3^/µL) were measured using a Sysmex^®^ analyzer (Sysmex Corporation, Kobe, Japan), which utilizes fluorescent flow cytometry with a semiconductor laser and hydrodynamic focusing.

#### 2.3.3. Flow Cytometry

Immunophenotyping was conducted following flow cytometry procedures from whole blood samples, which were carried out using sterile, single-use Falcon^®^ 12 × 75 mm polystyrene tubes with caps of 5 mL (Cat. No. 349202, Corning Science México S.A. de C.V., Reynosa, Mexico). After lysing red blood cells with 1:10 diluted FACS™ Lysing Solution (10× concentrate) with deionized water (Cat. No. 349202; Becton, Dickinson and Company, Franklin Lakes, NJ, USA), monoclonal antibodies conjugated with specific fluorochromes from BD^®^ Biosciences reagents (Becton, Dickinson and Company, Franklin Lakes, NJ, USA) were added: CD3 (SK7; T-cell antigen receptor complex; Cat. No. 345763, FITC), CD4 (SK3; Cat. No. 345769, PE), CD8 (SK1; Cat. No. 345772, FITC), CD45 (2D1; Cat. No. 345809, PerCP), CD56 (MY31; Cat. No. 345810, PE), CD69 (L78; Cat. No. 340560, APC), CD19 (SJ25C1; Cat. No. 345791, APC), and BD Tritest™ CD3/CD16 + CD56/CD45 (Cat. No. 342411). Cells were then incubated at room temperature in the dark with monoclonal antibodies for 30 min and washed with BD^®^ CellWASH^TM^ (Cat. No. 349524, Becton, Dickinson and Company, Franklin Lakes, NJ, USA). The BD FACSCalibur™ flow cytometer analyzer (Becton, Dickinson and Company, Franklin Lakes, NJ, USA) was used, and data interpretation was performed with BD CellQuest™ Pro, The Premier Acquisition and Analysis Software, version 6.0 (Becton, Dickinson and Company, Franklin Lakes, NJ, USA), and expressed as the % of total peripheral blood mononuclear cells (PBMCs). Using flow cytometry, the following cell populations were defined based on their phenotypic traits:

- Total T lymphocytes (TTLys): CD45^+^CD3^+^;
- Early activated T lymphocytes (EATLys): CD45^+^3^+^69^+^;
- Total NK lymphocytes (TNKLys): CD45^+^CD3^−^CD56^+^CD16^+^;
- NK^dim^ lymphocytes (NK^dim^): low expression of CD56^+^ on flow cytometry;
- NK^bright^ lymphocytes (NK^bright^): high expression of CD56^+^ on flow cytometry;
- B lymphocytes (BLys): CD45^+^CD3^−^CD19^+^;
- T helper lymphocytes (ThLys): CD45^+^CD3^+^CD4^+^;
- T cytotoxic lymphocytes (TcLys): CD45^+^CD3^+^CD8^+^.

#### 2.3.4. Multiplex Bead-Based Immunoassay

Serum concentrations of tumor necrosis factor-α (TNF-α) were determined using a multiplex bead-based immunoassay, employing Luminex^®^ xMAP^®^ technology and the MILLIPLEX^®^ Human Adipokine Magnetic Bead Panel 2 kit (Cat. No. HADK2MAG-61; Merck KGaA, Darmstadt, Germany; MilliporeSigma, Seattle, WA, USA). The parameters were quantified using the FLEXMAP 3D^®^ analyzer (Luminex Corporation, Austin, TX, USA, a DiaSorin Company, Saluggia, Italy), analyzed with xPONENT^®^ software version 4.3, and reported in pg/mL.

#### 2.3.5. Enzyme-Linked Immunosorbent Assay

The enzyme-linked immunosorbent assay (ELISA) method was utilized to quantify serum concentrations of interferon-γ (IFN-γ). The Quantikine^®^ ELISA Human IFN-γ kit (Cat. No. DIF50C/SIF50C/PDIF50C, R&D Systems, Inc., Minneapolis, MN, USA) and ELISA Dynex DSX analyzer (DYNEX^®^ TECHNOLOGIES, Chantilly, VA, USA) were employed. The results were reported in pg/mL.

### 2.4. Statistical Methods

The statistical analysis included descriptive statistics (frequency, percentage, mean, median, standard deviation) and inferential statistics. The Shapiro–Wilk test was applied to determine the distribution of the analyzed data series. The one-way analysis of variance test (ANOVA), a parametric test, was applied along with the Bonferroni test for multiple comparisons. Additionally, the Kruskal–Wallis test, a nonparametric test, was used along with the Dunn test for multiple comparisons. The Pearson test was applied to evaluate the correlation (measure of the strength of association) between quantitative variables, and the Chi-square test was used to determine the association between qualitative variables. In addition, a multivariate linear regression analysis was applied to evaluate the relationship between the independent variables and the dependent variable, controlling for the effects of other factors. The results were reported as regression coefficients (β) with 95% confidence intervals. Significance tests were conducted for each coefficient to determine their statistical relevance. The significance threshold chosen for the *p*-value was 0.05. The statistical analysis was performed using the GraphPad Prism software trial version 7.0.

## 3. Results

### 3.1. General Cohort Characterization

Out of 167 subjects enrolled in the study, 122 met the eligibility criteria. They were classified into three groups according to BMI: normal-weight (NW, 33 subjects, 27.05%, BMI 18.50–24.99), overweight (OW, 51 subjects, 41.80%, BMI 25.00–29.99), and obese (OB, 38 subjects, 31.15%, BMI ≥ 30.00). The general description of the cohort is detailed in Table 1. The age was slightly higher in the OB group (30 years old) compared to the NW and OW groups (27 years old for both groups). The sex distribution was approximately similar in the OW group (47.06% females vs. 52.94% males), compared to the OB group (34.21% females vs. 65.79% males) and the NW group (84.85% females vs. 15.15% males).

Additionally, the anthropometric measurements were analyzed and are detailed in Table 2. Overall, the adipometric indices showed a significant constant increase from the NW group to the OB group, including BMI, TBFM, VFL, WC, HC, WHR, and WHtR (*p* < 0.0001 for all indices).

### 3.2. Lymphocyte Phenotypes and Cytokine Concentrations

The WBC, Ly absolute count, and Ly subpopulations expressions are presented in Table 3. The analysis of data reveals significant differences in WBC across different BMI categories. Specifically, the mean WBC was significantly higher in the OW and OB groups compared to the NW group (7.07 for OB and 6.18 for OW vs. 5.92 for NW, *p* < 0.0001). Conversely, the Ly absolute count did not show a statistically significant difference across the BMI categories (*p* = 0.2025). Although there is a trend towards higher Ly counts in the OW and OB groups compared to the NW group, these differences are not statistically significant (2.33 for OB and 2.15 for OW vs. 2.11 for NW).

The first population of lymphocytes analyzed consisted of TTLy, which showed no significant differences between groups (*p* = 0.5992). The lowest proportion was observed in the OB group (72.35) and the highest in the OW group (74.60). However, significant differences were observed in the percentage of EATLy, with the NW group exhibiting the highest expression of CD69^+^ (3.10), followed by OW (1.10) and OB (0.85), respectively. The additional post hoc test (Dunn’s multiple comparison test) revealed significant differences in NW vs. OW (*p* < 0.0001) and NW vs. OB (*p* < 0.0001), but not in OW vs. OB (*p* = 0.0670).

Furthermore, the helper and cytotoxic subsets of TLy were divided and analyzed. No significant differences were observed between the groups for the individual subsets of ThLy and TcLy, nor in the CD4/CD8 ratio (*p* = 0.5147; *p* = 0.4261; *p* = 0.4532). However, the NW group presented a higher ThLy population (46.00), followed by the OB (43.40) and OW groups (43.30). Regarding the TcLy population, the OW group showed the highest expression (33.20), followed by the NW (32.00) and OB groups (31.50). Corresponding to the variations in ThLy and TcLy populations, the CD4/CD8 ratio was lowest in the OW group (1.28), followed by the NW (1.32) and OB groups (1.35).

The second lymphocyte population studied was BLy, whose mean expression was found to be significantly different between the groups (9.80 for NW; 9.07 for OW; 11.31 for OB; *p* = 0.0085). Furthermore, Bonferroni’s multiple comparison tests identified a significantly higher BLy population in the OB group compared to the OW group (11.00 vs. 8.90, *p* = 0.0019), while the differences in NW vs. OW (*p* = 0.3323) and NW vs. OB (*p* = 0.0673) were not significant.

The NKLys were classified into total, dim, and bright categories. The expression of TNKLy was found to be nonsignificantly lower in the OB group (15.15) compared to the NW (15.30) and OW groups (16.70) (*p* = 0.7828). Additionally, both subsets of NKLy, NK^dim^ and NK^bright^, showed no significant differences between groups (*p* = 0.8903; *p* = 0.6325), with a decrease in expression for NK^dim^ (14.40 for NW vs. 13.40 for OW vs. 13.10 for OB) and a constant expression for NK^bright^ (0.80 for all three groups).

The serum concentration of TNF-α was significantly higher in the OW group (5.22, *p* < 0.0001) compared to the NW and OB groups (3.44 and 4.50, respectively). Dunn’s test confirmed that all pairwise comparisons were statistically significant: NW vs. OW (*p* < 0.0001), NW vs. OB (*p* = 0.0040), and OW vs. OB (*p* = 0.0124). Although IFN-γ did not show significant differences overall (*p* = 0.0680), an increase was observed from the NW group (18.74) to the OW (21.78) and OB (22.37) groups. Furthermore, Dunn’s test highlighted a significant difference only between the NW and OB groups (*p* = 0.0380).

### 3.3. Correlation Between Lymphocyte Subsets and Adiposity Indices 

Additionally, we analyzed the correlations between lymphocyte subtypes and the adiposity indices, as presented in Table 4. For a comprehensive interpretation of the data, each lymphocyte population is analyzed separately with all adiposity indices.

TTLy exhibited two statistically insignificant correlations: it correlated negatively with BMI (r = −0.0275; *p* = 0.7634), TBFM (r = −0.0056; *p* = 0.9506), and VFL (r = −0.0085; *p* = 0.9258) and positively with WHR (r = 0.0095; *p* = 0.9165) and WHtR (r = 0.0305; *p* = 0.7381).

On the other hand, the EATLy (CD69^+^) population showed an inversely proportional relationship with all adiposity indices, significantly decreasing with increasing body adiposity. EATLy negatively correlates with all indices: BMI (r = −0.3741; *p* < 0.0001), WHR (r = −0.3339; *p* = 0.0002), WHtR (r = −0.3443; *p* = 0.0001), TBFM (r = −0.3113; *p* = 0.0005), and VFL (r = −0.3289; *p* = 0.0002).

Similar to EATLy, the ThLy population also decreases with increasing body adiposity. Consequently, only nonsignificant negative correlations were found between ThLy and all adiposity indices: BMI (r = −0.1206; *p* = 0.1856), WHR (r = −0.1348; *p* = 0.1388), WHtR (r = −0.1371; *p* = 0.1321), TBFM (r = −0.0437; *p* = 0.6321), and VFL (r = −0.0166; *p* = 0.8558).

On the contrary, TcLy mostly positively correlated with the adiposity indices (*p* > 0.05), except for TBFM (r = −0.0429; *p* = 0.6388) and VFL (r = −0.0424; *p* = 0.6422). Although there were some divergent correlations between ThLy and TcLy vs. adiposity indices, the CD4/CD8 ratio showed only positive correlations with all indices (*p* > 0.05).

All studied correlations between BLy and the adiposity indices were positive; however, none were significant (*p* > 0.05). The strongest correlation, according to the *p*-values, was observed between BLy and TBFM (r = 0.1770; *p* = 0.0512).

Additionally, all NK subsets showed an inverse relationship with all adiposity indices, and all these relationships were insignificant (*p* > 0.05). TNKLy correlated most strongly with WHtR (r = −0.1062; *p* = 0.2445) and least with WHR (r = −0.0004; *p* = 0.9960). However, NK^dim^ showed the strongest correlation with TBFM (r = −0.1023; *p* = 0.2622), while NK^bright^ had the strongest correlation with TBFM (r = −0.0762; *p* = 0.4037).

### 3.4. Correlation Between Lymphocyte Subsets and IFN-γ and TNF-α

**Figure 1:**
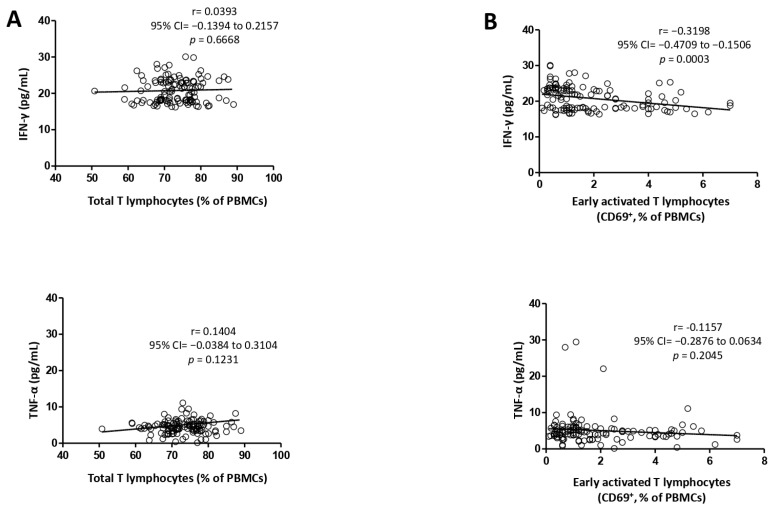
Correlation between total T lymphocytes (**A**) and early activated T lymphocyte populations (**B**) with IFN-γ and TNF-α. IFN-γ—interferon-γ; TNF-α—tumor necrosis factor-α; 95%CI—95% confidence interval; PBMCs—peripheral blood mononuclear cells. All correlations were analyzed using the Pearson test.

**Figure 2:**
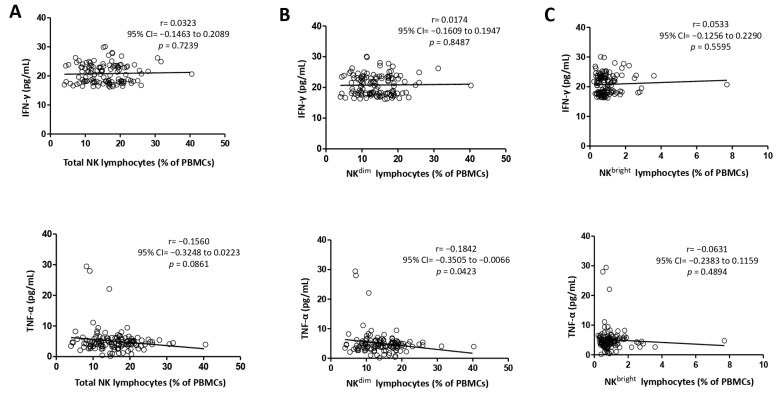
Correlation between NKLy subsets (**A**)—Total NK lymphocytes; (**B**)—NK^dim^ lymphocytes; (**C**)—NK^bright^ lymphocytes) and IFN-γ and TNF-α. IFN-γ—interferon-γ; TNF-α—tumor necrosis factor-α; 95%CI—95% confidence interval; PBMCs—peripheral blood mononuclear cells. All correlations were analyzed using the Pearson test.

The relationship between cytokine serum concentrations and lymphocyte populations was investigated, as illustrated in Figure 1 and Figure 2. TTLy showed a positive correlation with both cytokines, IFN-γ (r = 0.0393; *p* = 0.6668) and TNF-α (r =0.1404; *p* = 0.1231). Conversely, EATLy exhibited a negative correlation with both cytokines, significant with IFN-γ (r = −0.3198; R2= 0.10; *p* = 0.0003) and nonsignificant with TNF-α (r = −0.1157; R2= 0.01; *p* = 0.2045). Furthermore, the values of the coefficient of determination (R2) suggest that only 10% of the variability between the EATLy and INF-γ is explained by their relationship, with the remaining 90% indicating that other factors may be influencing the results.

Furthermore, TNF-α serum concentration correlated negatively with all NKLy subsets (Figure 2): TNKLy (r = −0.1560), NK^dim^ (r = −0.1842), and NK^bright^ (r = −0.0631), respectively. The significance cut-off for the *p*-value was reached only for the correlation between TNF-α and NK^dim^ (*p* = 0.0423). Additionally, all three NKLy populations showed a positive correlation with IFN-γ, but none were significant (*p* > 0.05): TNKLy (r = 0.0323), NK^dim^ (r = 0.0174), and NK^bright^ (r = 0.0533), respectively.

Next, a multivariate linear regression analysis was performed (Table 5), which revealed that an adjusted R2 of 0.9% of the variation in TNF-α values is explained by the variation in the predictors: EATLy, TTLy, NK^dim^, NK^bright^ and TNKLy. The ANOVA showed *p* > 0.05, indicating that the model is not statistically significant. On the other hand, the adjusted R2 = 7.7% of the variation in IFN-γ values is explained by the variation of the same predictors and associated with an ANOVA, *p* < 0.05 (*p* = 0.0136), indicating that the model is statistically significant. For EATLy, the coefficient β is −0.637, meaning that for each one-unit increase in the EATLy value, IFN-γ levels will decrease by 0.637, which is statistically significant.

### 3.5. Correlation Between Age and TNKLy, NK^dim^, and NK^bright^ Frequency

**Figure 3:**
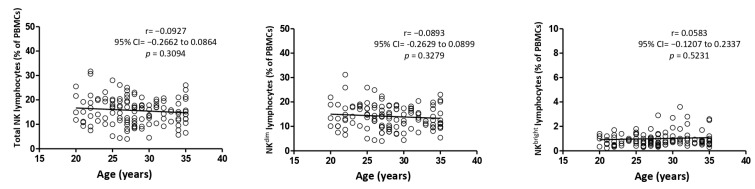
Correlation between NKLy populations and age. IFN-γ—interferon-γ; TNF-α—tumor necrosis factor-α; 95%CI—95% confidence interval; PBMCs—peripheral blood mononuclear cells. All correlations were analyzed using the Pearson test.

We also examined the influence of age on NKLy subsets, as presented in Figure 3. All correlations between age and NK lymphocyte populations were not significant (*p* = 0.3094 for TNKLy; *p* = 0.3279 for NK^dim^; *p* = 0.5231 for NK^bright^). However, a negative correlation was observed between age and TNKLy (r = −0.0927) and NK^dim^ (r = −0.0893), as well as a positive correlation with NK^bright^ (r = 0.0583).

## 4. Discussion

In this study, we demonstrated that in healthy adults aged 20–35, the baseline immunological profile of peripheral blood is influenced by excessive adipose tissue. While our findings are partially consistent with previous research, we also identified some results that diverge from those reported in the literature. Due to the specificity of this study’s inclusion criteria and the potential influence of various factors on the immune profile, we aimed to compare the results with studies that employed similar inclusion criteria whenever possible.

Notably, a significant upward gradient in WBC across BMI categories was observed from the beginning (5.92 for NW, 6.18 for OW, and 7.07 for OB; *p* < 0.0001). Although all values remain within the reference range, this trend suggests that increasing BMI is associated with higher WBC counts, potentially indicating a systemic inflammatory or immune response related to excess body weight. The trend was also consistent in the Ly absolute count, although the increase was not statistically significant (2.11 for NW, 2.15 for OW, and 2.33 for OB; *p* = 0.2025). The results converge with findings from a study conducted in Mexico involving a similar population of young adults with a mean age of 26.4 ± 6.8 years. This study reported a significant increase in total Ly as BMI increased [12]. A second study conducted in Mexico with a mean age of 34.3 ± 8.8 years similarly observed a significant increase in WBC within the reference range as the total body fat percentage increased (*p* = 0.013) [13]. Moreover, a study involving healthy women with a mean age of 35 years reported a significantly higher WBC in OB compared to the non-OB group (6.4 ± 0.3 vs. 4.4 ± 0.3, *p* = 0.035). Interestingly, a significant positive correlation was observed between WBC and triglycerides (r = 0.2, *p* = 0.03) as well as the atherogenic index of plasma (r = 0.3, *p* = 0.028) [14].

Routine lymphogram assessments typically do not focus on NKLys, a population that is often underinvestigated. However, NKLys are part of the innate immune system and are primarily responsible for eliminating cancerous and virus-infected cells. Over time, it has been demonstrated that their role is much more complex. NKLys are also involved in resolving inflammation, antibacterial effects, removing senescent cells, and modulating the adaptive immune response [15]. Morphologically, NKLys are large granular lymphocytes equipped with a detection system comprising various cell surface activating and inhibitory receptors. Upon activation, NKLys release their cytolytic granules and secrete cytokines such as IFN-γ and TNF-α. NKLys comprise approximately 10–20% of PBMCs and are characterized by their CD3^−^CD56^+^ expression pattern. However, flow cytometry analysis divides the NKLy population into two distinct subtypes of CD56^+^ with unique immunophenotypes and functions: low expression defined as CD56^dim^ (NK^dim^), and high expression noted as CD56^bright^ (NK^bright^), respectively [16]. NK^dim^ cells lose their cytokine responsiveness and present a direct cytotoxic function characterized by a higher concentration of perforin and granzyme within their intracytoplasmic granules. Conversely, NK^bright^ cells are primarily involved in the production of proinflammatory cytokines [17].

NK^dim^ cells form 90% of the NK cell population in peripheral blood, while NK^bright^ cells dominate various human tissues such as the adipose tissue, lung, uterus, liver, lymph nodes, and intestine [11]. The tissue-specific microenvironment significantly influences the phenotype predominance of NKLy. Research suggests that obesity is associated with impaired degranulation and cytokine production capacity of NKLy, potentially increasing the risk of cancer and chronic inflammatory diseases [18]. In addition, these impairments of NKLy are maintained in cases of induced obesity, resulting in increased IFN-γ secretion in VAT and influencing macrophage polarization [9]. However, this process seems to be reversible following weight loss [19].

The Mexican study reported a significant decrease in the TNKLy population across BMI categories (19.5 for NW, 15.6 for OW, and 14.9 for OB, *p* = 0.009). Furthermore, following classification based on VAT, the population of TNKLy was found to be lower in the group with increased VAT [12]. Another study not only confirmed previous findings that TNKLys are significantly reduced in OB subjects compared to NW (7.6 vs. 16.6, *p* = 0.0008), but also investigated the cytotoxic function against K562 target cells, which was found to be significantly lower in the OB group (*p* = 0.04) [20]. In contrast to these results, our findings suggest a sinusoidal pattern, but still with a lower expression, also observed in the OB group: 15.30 for NW, 16.70 for OW, and 15.15 for OB. The inverse relationship between peripheral blood TNKLy and adiposity indices may be attributed to the migration of these cells into adipose tissue in response to the expression of local tissue-specific environmental signals. In our study, when we analyzed the correlation with anthropometric and body composition indices, TNKLy showed a nonsignificant negative correlation with all measured parameters. On the contrary, a study involving Korean young adults (mean age of 26 years old), which defined the OB group based on a BMI ≥ 25, reported a nonsignificant higher TNKLy expression in OB [21].

Our analysis of NKLy subset expression aligns with previous studies, showing that NK^dim^ constitutes 80–90% of BMI group-specific TNKLy in peripheral blood, with higher expression in the NW group. However, when comparing group-by-group NK^dim^ expression, a nonsignificant decline was observed from NW to OB. The NK^bright^ accounts for 4–5% of BMI group-specific TNKLy in peripheral blood, with higher expression in the OB group. Moreover, NK^bright^ showed a stable mean expression (0.80, *p* = 0.6325) across group-by-group comparison. The second study from Mexico observed a similar downward trend in the percentage expression of TNKLy as total body fat percentage increased, though this change did not reach statistical significance (*p* = 0.105) [13].

Furthermore, our study analyzed the impact of age on the expression of NKLy subsets. Beyond changes in cell numbers, human aging is associated with a decline in NKLy function, particularly affecting direct cytotoxicity and cytokine secretion, which predisposes the older population to an increased risk and frequency of cancer and viral infections [15,22]. Increasing age was correlated with an increase in TNKLy and NK^dim^ cells and a decrease in NK^bright^ cells in a healthy Korean population aged 20–76 years [23]. In Germany (19–67 years old), a lower percentage but higher absolute count of NK^dim^ cells was reported in the elderly group, while in the United Kingdom, only the absolute count of NK^bright^ cells in peripheral blood declined significantly (*p* = 0.0004) with age [24,25]. On the contrary, in our study, a negative correlation was observed between age and both TNKLy (r = −0.0927) and NK^dim^ (r = −0.0893), with a positive correlation with NK^bright^ (r = 0.0583). The discrepancy may be explained by the age range of our cohort (20–35 years), whereas, in previously cited studies, this age group was only included in the younger population and compared to significantly older patients. Furthermore, a study comprising an age range of 51 to 68 years reported an increase in NK^bright^ and a decrease in NK^dim^ in OB individuals, while the overall frequency of TNKLy remained unchanged (3.9 for NW vs. 3.8 for OB). Additionally, NK^bright^ in the OB group showed elevated expression of NKG2D (activating NK cell receptor) and intracellular IFN-γ. Conversely, NK^dim^ cells in the obese group showed reduced levels of both NKG2D and IFN-γ [10].

Immunocompetence is also maintained by BLys, which are integral to the humoral immune response and the establishment of long-term immune protection. Conversely, the development and regulation of this immune memory are managed by various classes of TLy, each with distinct roles. In our study, the OB group exhibited a significant higher BLy population (11.00, *p* = 0.0085) compared to the other groups. Furthermore, this cellular distribution is supported by positive correlations with all adiposity indices, including BMI, WHR, WHtR, TBFM, and VFL. Compared to our findings, a study reported a nonsignificant increase in the frequency of BLy cells (7.5 vs. 9.4, *p* = 0.4367) but a significant decrease in TTLy cells (68.3 vs. 54.4, *p* = 0.0145) in the OB group [10]. In this study, the TTLy population did not show a significant difference between groups (71.40 for NW, 74.60 for OW, 72.35 for OB; *p* = 0.5992). However, the EATLy subpopulation was significantly higher in the NW group compared to the excessive adiposity groups (3.10 vs. 1.10/0.85; *p* < 0.0001). Consistent with our findings, a second study from Mexico reported that both the absolute count and percentage expression of TTLy increased with rising BMI. However, only the absolute count reached statistical significance (*p* < 0.001), while the percentage expression showed an upward trend that was not statistically significant (*p* = 0.095). Furthermore, contrary to our results, they reported a significant decrease in the percentage expression of the BLy population with an increase in the total body fat percentage, while the absolute count showed a nonsignificant increase [13]. In addition to comparing the immunological profiles of peripheral blood and adipose tissue, a study highlighted that bone marrow cellularity is impacted by excessive fat distribution. Moreover, research has shown that in individuals who tested negative for Cytomegalovirus, the frequency of BLy and ThLy in the bone marrow positively correlates with BMI [26].

CD69 (a type II C-lectin receptor) is a marker of early activation in most leukocytes, as it is expressed on the cell within 30 to 60 min upon activation [27]. Its expression is also observed in infiltrated Ly in various chronic inflammatory diseases, including systemic lupus erythematosus, allergic asthma, arthritis, and the pathogenesis of type 2 diabetes [28,29]. A previous study defined CD69 as a marker of tissue retention immune cells by inhibiting sphingosine-1-phosphate receptor 1 [27]. To date, there is a gap in the medical literature regarding the correlation between CD69 expression on TTLy and adipose tissue in healthy young adults. However, a study involving obese subjects with prediabetes or type 2 diabetes undergoing weight loss and energy restriction reported that CD69 expression on TTLy (EATLy) in peripheral blood decreases following BMI reduction [29]. This study’s analysis revealed a significantly decreased expression of this marker in the OB group compared to the NW group (0.85 vs. 3.10, *p* < 0.0001). Additionally, EATLy showed a significant negative correlation with all adiposity indices, including BMI, WHR, WHtR, TBFM, and VFL.

In our study, the frequency of ThLy did not show any significant differences between groups (*p* = 0.5147), but the mean expression was decreased in the excessive adipose tissue groups compared to the NW group (43.30/43.40 vs. 46). These data suggest that ThLy decreases as adipose tissue quantity increases, a finding supported by all negative correlations between ThLy and all studied adiposity indices: BMI, WHR, WHtR, TBFM, and VFL. However, TcLy expression was highest in the OW group, although the median values show minimal changes between groups (33.20 for OW, 31.50 for OB, and 32 for NW, *p* = 0.7202). The TcLy frequency presented two directions when correlated nonsignificantly with adiposity indices: it increases as BMI, WHR, and WHtR increase and decreases as TBFM and VFL increase. In line with our findings, the Korean study reported a significantly lower TcLy population in OB subjects compared to NW individuals (23.3 ± 1.5 in NW vs. 19.4 ± 1.2 in OB, *p* = 0.044). Although the results were not statistically significant, the ThLy expression in that study contrasted with ours, showing higher ThLy levels in the OB group [21]. Another study reported a significant decrease in both the absolute count (*p* = 0.045) and percentage (*p* = 0.001) of TcLy in OB groups compared to NW. In contrast to our findings, this study also observed a significant increase in ThLy with increasing adiposity, both in absolute count (*p* < 0.001) and percentage (*p* = 0.001) [13]. Furthermore, another study reported a significantly higher frequency of ThLy (74.5 vs. 62.6, *p* = 0.004) and a lower frequency of TcLy (22.3 vs. 31.7, *p* = 0.016) in the OB group compared to the NW group. Additionally, when evaluating the surface expression of CD28, CD95, and CD62L as markers of activation, apoptosis, and migration, only a significant increase in CD95^+^ expression among TcLys (71.9 vs. 58.6, *p* = 0.034) was observed. Correlation analysis with BMI revealed a positive correlation for ThLy (R2 = 0.462, *p* = 0.001) and TcLyCD95+ (R2 = 0.154, *p* = 0.088), while TcLy showed a negative correlation (R2 = −0.367, *p* = 0.005) [30]. These discrepancies compared to our study may be attributed to differences in inclusion criteria, as the previous study did not restrict enrollment to healthy individuals (including patients with comorbidities) and had a wider age range (mean age of 55 years for the OB group vs. 34 years for the NW group).

Additionally, we analyzed the link between the common proinflammatory cytokines IFN-γ and TNF-α and the peripheral blood lymphocyte subsets. Since several types of lymphocytes secrete these two cytokines, we analyzed the link between each subtype and the serum concentration separately. Depending on the migration direction of the cell subset, the correlation can be observed from a mirrored perspective. In our study, both cytokine serum concentrations increased as the peripheral blood TTLy frequency increased (r = 0.0393, *p* = 0.6668 for IFN-γ; r = 0.1404, *p* = 0.1231 for TNF-α). On the other hand, there was a marked negative correlation between the peripheral blood EATLy and the IFN-γ serum concentration (r = −0.3198, *p* = 0.003). A nonsignificant negative correlation was found between the peripheral blood EATLy and the serum concentration of TNF-α (r = −0.1157, *p* = 0.2045). A study used real-time PCR analysis to compare the expression level of TTLy for several cytokines in peripheral blood vs. SAT. A significantly higher transcript level in VAT TTLy was found for IFN-γ, TNF-α, RANTES, IL-2, and perforin-1 compared to blood TTLy [31].

Among the limitations of this study, we can enumerate the relatively small cohort size and the unbalanced sex distribution in the NW group, which may limit the extensibility of the results. Increasing the study groups and a balanced sex distribution may provide clearer and more statistically significant results. A significant impact may be attributed to sex ratios between BMI groups, as gender is a known risk factor for obesity. This disparity may introduce bias into the analysis, particularly in Ly count and the CD4/CD8 ratio [32,33]. Although the literature reports higher Ly absolute counts in females, in our study, the NW group, which was predominantly composed of females, had the lowest lymphocyte count compared to the other groups. Additionally, the design of the study limits the establishment of causal relationships between excessive adipose tissue and alterations in the immunological profile. Furthermore, by focusing solely on CD69^+^ expression in TTLy, the immunological links with other lymphocyte subpopulations may be missed. Including additional surface markers could offer a complete understanding of the immune picture in the context of obesity.

Based on these limitations and the study’s findings, future research should consider a more detailed classification of lymphocyte subsets and explore CD69^+^ expression within NKLy subpopulations. Expanding the age range of participants could also enhance the comparison degree of the results. On the other hand, additional markers of activation, apoptosis, secretion, and migration can be taken into consideration. Additionally, parallel analyses of cellular expression and cytokine concentrations in both peripheral blood and adipose tissue (SAT and VAT) could provide a complete understanding of the immunological changes in obesity.

## 5. Conclusions

In summary, the close interrelationship between adipose tissue and the immune system has been observed in apparently healthy adults who are 20–35 years old. Multiple mechanisms are likely to act in a synergistic manner to influence these changes.

The data reveal a slightly, insignificantly impaired NKLy phenotype and subset alterations in OW and OB individuals. Although a statistically significant *p*-value was not found, within this relatively small age range, age appeared to influence the NKLy population, showing a negative effect on TNKLy and NK^dim^ and a positive effect on NK^bright^.

TTLy showed a nonsignificant positive correlation with both IFN-γ and TNF-α, while EATLy exhibited an opposite negative correlation with both, revealing a significant correlation only with IFN-γ. The correlation analysis of NKLy subpopulations revealed a consistent negative correlation with TNF-α across all NKLy subpopulations, though significant only for NK^dim^. Conversely, all NKLy subpopulations had a consistent positive but nonsignificant correlation with IFN-γ.

After all, can obesity be classified as an immune disease? To better understand and define the immunological profile of peripheral blood and the mechanisms underlying the inflammatory response of adipose tissue, larger studies involving healthy populations across all age groups are recommended.

## Figures and Tables

**Table 1:** General description of the study cohort.

Criteria		Normal Weight	Overweight	Obese
Age	Mean ± SD (Median)	28.06 ± 3.55	27.45 ± 4.32	28.87 ± 4.94
Sex	Female—n (%)	28.00 (84.85%)	24.00 (47.06%)	13.00 (34.21%)
	Male—n (%)	5.00 (15.15%)	27.00 (52.94%)	25.00 (65.79%)

SD—standard deviation; n—number.

**Table 2:** Group description of the anthropometric measurements according to BMI classification.

Criteria	Normal Weight (n = 33) Mean ± SD (Median)	Overweight (n = 51) Mean ± SD (Median)	Obese (n = 38) Mean ± SD (Median)	*p*-Value	Post Hoc Test *p* < 0.05
Body mass index	21.09 ± 1.74 (21.10)	27.33 ± 1.25 (27.50)	35.89 ± 4.86 (34.60)	<0.0001 *	Yes ‡
Total body fat mass (kg)	13.30 ± 3.67 (13.10)	24.08 ± 5.68 (24.10)	40.86 ± 14.82 (35.75)	<0.0001 *	Yes ‡
Visceral fat level	1.84 ± 0.90 (2.00)	5.94 ± 2.07 (5.00)	12.47 ± 5.57 (12.50)	<0.0001 *	Yes ‡
Waist circumference (cm)	72.33 ± 7.71 (72.00)	91.41 ± 8.32 (92.00)	108.80 ± 14.22 (107.00)	<0.0001 **	Yes §
Hip circumference (cm)	97.15 ± 4.72 (97.00)	107.50 ± 5.18 (107.00)	121.20 ± 9.13 (120.00)	<0.0001 **	Yes §
Waist-to-hip ratio	0.74 ± 0.06 (0.71)	0.84 ± 0.08 (0.84)	0.89 ± 0.09 (0.89)	<0.0001 *	Yes ‡
Waist-to-height ratio	0.43 ± 0.03 (0.42)	0.52 ± 0.03 (0.52)	0.62 ± 0.06 (0.60)	<0.0001 *	Yes ‡

SD—standard deviation; * Kruskal–Wallis test; ** one-way analysis of variance (ANOVA); ‡ Dunn test; § Bonferroni test.

**Table 3:** Distribution of complete blood count parameters, lymphocyte subsets, and serum cytokine concentrations of the study groups according to BMI.

Criteria	Normal Weight (no = 33) Mean ± SD (Median)	Overweight (no = 51) Mean ± SD (Median)	Obese (no = 38) Mean ± SD (Median)	*p*-Value	Post Hoc Test *p* < 0.05
Complete Blood Count					
White blood cells (×10^3^ cells/µL)	6.07 ± 1.38 (5.92)	6.56 ± 1.34 (6.18)	7.46 ± 1.72 (7.07)	<0.0001 *	Yes ‡
Lymphocyte absolute count (×10^3^ cells/µL)	2.13 ± 0.53 (2.11)	2.28 ± 0.53 (2.15)	2.38 ± 0.69 (2.33)	0.2025 *	No ‡
Flow cytometry					
Total T lymphocytes (% of PBMCs)	73.20 ± 6.81 (71.40)	73.62 ± 6.05 (74.60)	72.52 ± 6.25 (72.35)	0.5992 *	No ‡
Early activated T lymphocytes (% of PBMCs)	3.19 ± 1.73 (3.10)	1.62 ± 1.43 (1.10)	1.22 ± 1.17 (0.85)	<0.0001 *	OW vs. OB NS ‡
T helper lymphocytes (% of PBMCs)	44.82 ± 7.17 (46.00)	42.74 ± 7.37 (43.30)	42.48 ± 8.70 (43.40)	0.5147 *	No ‡
T cytotoxic lymphocytes (% of PBMCs)	33.12 ± 5.33 (32.00)	34.65 ± 8.23 (33.20)	33.10 ± 10.09 (31.50)	0.4261 *	No ‡
CD4/CD8	1.40 ± 0.37 (1.32)	1.30 ± 0.39 (1.28)	1.42 ± 0.55 (1.35)	0.4532 **	No §
B lymphocytes (% of PBMCs)	9.80 ± 3.51 (9.50)	9.07 ± 3.20 (8.90)	11.31 ± 3.29 (11.00)	0.0085 **	NW vs. OW NS NW vs. OB NS §
Total NK lymphocytes (% of PBMCs)	15.53 ± 6.08 (15.30)	16.09 ± 6.25 (16.70)	15.19 ± 5.81 (15.15)	0.7828 *	No ‡
NK^dim^ lymphocytes (% of PBMCs)	14.17 ± 5.49 (14.40)	14.31 ± 6.17 (13.40)	13.71 ± 4.82 (13.10)	0.8903 *	No ‡
NK^bright^ lymphocytes (% of PBMCs)	1.15 ± 1.30 (0.80)	0.89 ± 0.52 (0.80)	1.02 ± 0.67 (0.80)	0.6325 *	No ‡
Cytokines					
TNF-α (pg/mL)	3.44 ± 1.43 (3.44)	6.49 ± 5.43 (5.22)	4.54 ± 1.66 (4.50)	<0.0001 *	Yes ‡
IFN-γ (pg/mL)	19.48 ± 2.46 (18.74)	21.04 ± 3.12 (21.78)	21.59 ± 3.76 (22.37)	0.0680 *	No ‡

PBMCs—peripheral blood mononuclear cells; NW—normal weight; OW—overweight; OB—obese; NS—not significant; SD—standard deviation; * Kruskal–Wallis test; ** one-way analysis of variance (ANOVA); ‡ Dunn test; § Bonferroni test.

**Table 4:** (A) Correlations between lymphocyte subpopulations with body mass index, waist-to-hip ratio, and waist-to-height ratio. (B) Correlation between lymphocyte subpopulations with total body fat mass and visceral fat level.

(A)						
**Criteria**	**BMI**		**WHR**		**WHtR**	
	**r (95% CI)**	*p* *	**r (95% CI)**	*p* *	**r (95% CI)**	*p* *
Total T lymphocytes (% of PBMCs)	−0.0275 (−0.2043 to 0.1510)	0.7634	0.0095 (−0.1685 to 0.1871)	0.9165	0.0305 (−0.1480 to 0.2073)	0.7381
Early activated T lymphocytes (CD69^+^, % of PBMCs)	−0.3741 (−0.5175 to −0.2103)	<0.0001	−0.3339 (−0.4831 to −0.1660)	0.0002	−0.3443 (−0.4920 to −0.1773)	0.0001
T helper lymphocytes (% of PBMCs)	−0.1206 (−0.2922 to 0.0584)	0.1856	−0.1348 (−0.3053 to 0.0440)	0.1388	−0.1371 (−0.3074 to 0.0417)	0.1321
T cytotoxic lymphocytes (% of PBMCs)	0.0069 (−0.1711 to 0.1845)	0.9396	0.0094 (−0.1687 to 0.1869)	0.9180	0.0327 (−0.1459 to 0.2093)	0.7202
CD4/CD8	0.0418 (−0.1370 to 0.2180)	0.6471	0.0160 (−0.1622 to 0.1933)	0.8607	0.0312 (−0.1474 to 0.2079)	0.7327
B lymphocytes (% of PBMCs)	0.1336 (−0.0452 to 0.3042)	0.1423	0.0772 (−0.1019 to 0.2516)	0.3975	0.1335 (−0.0454 to 0.3041)	0.1427
Total NK lymphocytes (% of PBMCs)	−0.0606 (−0.2359 to 0.1184)	0.5067	−0.0004 (−0.1782 to 0.1774)	0.9960	−0.1062 (−0.2787 to 0.0730)	0.2445
NK^dim^ lymphocytes (% of PBMCs)	−0.0561 (−0.2316 to 0.1229)	0.5394	−0.0282 (−0.2051 to 0.1503)	0.7571	−0.0976 (−0.2708 to 0.0815)	0.2846
NK^bright^ lymphocytes (% of PBMCs)	−0.0529 (−0.2286 to 0.1261)	0.5627	−0.0559 (−0.2314 to 0.1231)	0.5408	−0.0492 (−0.2251 to 0.1297)	0.5899
(B)						
**Criteria**			**Total Body Fat Mass (kg)**		**Visceral Fat Level**	
			**r (95% CI)**	*p* *	**r (95% CI)**	*p* *
Total T lymphocytes (% of PBMCs)			−0.0056 (−0.1833 to 0.1723)	0.9506	−0.0056 (−0.1833 to 0.1723)	0.9506
Early activated T lymphocytes (CD69^+^, % of PBMCs)			−0.3113 (−0.4634 to −0.1413)	0.0005	−0.3113 (−0.4634 to −0.1413)	0.0005
T helper lymphocytes (% of PBMCs)			−0.0437 (−0.2199 to 0.1351)	0.6321	−0.0437 (−0.2199 to 0.1351)	0.6321
T cytotoxic lymphocytes (% of PBMCs)			−0.0429 (−0.2190 to 0.1359)	0.6388	−0.0429 (−0.2190 to 0.1359)	0.6388
CD4/CD8			0.0942 (−0.0850 to 0.2675)	0.3020	0.0942 (−0.0850 to 0.2675)	0.3020
B lymphocytes (% of PBMCs)			0.1770 (−0.0008 to 0.3440)	0.0512	0.1770 (−0.0008 to 0.3440)	0.0512
Total NK lymphocytes (% of PBMCs)			−0.0972 (−0.2704 to 0.0820)	0.2867	−0.0972 (−0.2704 to 0.0820)	0.2867
NK^dim^ lymphocytes (% of PBMCs)			−0.1023 (−0.2751 to 0.0769)	0.2622	−0.1023 (−0.2751 to 0.0769)	0.2622
NK^bright^ lymphocytes (% of PBMCs)			−0.0762 (−0.2507 to 0.1029)	0.4037	−0.0762 (−0.2507 to 0.1029)	0.4037

BMI—body mass index; WHR—waist-to-hip ratio; WHtR—waist-to-height ratio; PBMCs—peripheral blood mononuclear cells. PBMCs—peripheral blood mononuclear cells. * All correlations were performed with the Pearson test.

**Table 5:** Multivariate linear regression analysis of TNF-α and IFN-γ levels and their predictors.

	β	CI 95% for β	*p*-Value
TNF-α (pg/mL)			
Early activated T lymphocytes (CD69^+^, % of PBMCs)	−0.281	−0.710 to 0.149	0.198
Total T lymphocytes (% of PBMCs)	−0.029	−0.229 to 0.171	0.776
NK^dim^ lymphocytes (% of PBMCs)	−0.219	−0.609 to 0.172	0.271
NK^bright^ lymphocytes (% of PBMCs)	−0.043	−0.900 to 0.815	0.922
Total NK lymphocytes (% of PBMCs)	0.061	−0.258 to 0.380	0.704
IFN-γ (pg/mL)			
Early activated T lymphocytes (CD69^+^, % of PBMCs)	−0.637	−0.984 to −0.291	0.0004
Total T lymphocytes (% of PBMCs)	0.068	−0.093 to 0.230	0.403
NK^dim^ lymphocytes (% of PBMCs)	0.013	−0.302 to 0.329	0.933
NK^bright^ lymphocytes (% of PBMCs)	0.292	−0.401 to 0.985	0.406
Total NK lymphocytes (% of PBMCs)	0.047	−0.211 to 0.305	0.719

## Data Availability

Data are contained within the article.

## Abbreviations

BLy	B lymphocytes
BMI	body mass index
CLGI	chronic low-grade inflammation
EATLy	early activated T lymphocytes
HC	hip circumference
Ht	height
IFN-γ	interferon-γ
Ly	lymphocyte
NK^bright^	natural killer lymphocytes with a high expression of CD56^+^ on flow cytometry
NK^dim^	natural killer lymphocytes with a low expression of CD56^+^ on flow cytometry
NKLy	natural killer lymphocytes
NW	normal weight
OB	obese
OW	overweight
PB	peripheral blood
PBMCs	peripheral blood mononuclear cells
TBFM	total body fat mass
TcLy	T cytotoxic lymphocytes
ThLy	T helper lymphocytes
TNF-α	tumor necrosis factor-α
TNKLy	total NK lymphocytes
TTLy	total T lymphocytes
WBC	white blood cell count
WC	waist circumference
WHR	waist-to-hip ratio
WHtR	waist-to-height ratio
VFL	visceral fat level
